# Correction: Molecular snapshot of drug-resistant *Mycobacterium tuberculosis* strains from the Plateau State, Nigeria

**DOI:** 10.1371/journal.pone.0315611

**Published:** 2024-12-06

**Authors:** Zofia Bakuła, Valentine B. Wuyep, Łukasz Bartocha, Anna Vyazovaya, Eugene I. Ikeh, Jacek Bielecki, Igor Mokrousov, Tomasz Jagielski

An additional affiliation is missing for the seventh author. Igor Mokrousov is also affiliated with the Henan International Joint Laboratory of Children’s Infectious Diseases, Children’s Hospital Affiliated to Zhengzhou University, Zhengzhou, China.
