# Supplementary material for: Molecular snapshot of drug-resistant Mycobacterium tuberculosis strains from the Plateau State, Nigeria
Source: PLoS One. 2022 May 24;17(5):e0266837. doi: 10.1371/journal.pone.0266837 (PMC9129033; doi:10.1371/journal.pone.0266837)
Supplement: S1 File — (DOCX) [file pone.0266837.s001.docx]

**SUPPLEMENTARY MATERIAL**

**Molecular snapshot of drug resistant *Mycobacterium tuberculosis* strains from the Plateau State, Nigeria**

Zofia Bakuła^1^, Valentine B. Wuyep^2^ Łukasz Bartocha^1^, Anna Vyazovaya^3^, Eugene I. Ikeh^4^, Jacek Bielecki^1^, Igor Mokrousov^3^, Tomasz Jagielski^1^

^1^Department of Medical Microbiology, Institute of Microbiology, Faculty of Biology, University of Warsaw, Poland

^2^Plateau State College of Health Technology, Zawan, Jos, Nigeria

^3^Laboratory of Molecular Epidemiology and Evolutionary Genetics, St. Petersburg Pasteur Institute, St. Petersburg, Russia

^4^Department of Medical Microbiology, School of Medical and Health Sciences, College of Medical Sciences, University of Jos, Nigeria

**S1 Table. Patient place of living and ethnicity in different parts of the Plateau State.**

| **Category** | | **Part of the Plateau state (n/%)** | | |
| --- | --- | --- | --- | --- |
|  |  | **North, n=52** | **Center, n=11** | **South, n=4** |
| **Area** | **Urban, n=52** | 49/94.2 | 1/9.1 | 2/50 |
|  | **Rural, n=15** | 3/5.8 | 10/90.9 | 2/50 |
| **Ethnic group** | **Hausa, n=26** | 26/50 | - | - |
|  | **Berom, n=22** | 22/42.3 | - | - |
|  | **Ngas, n=8** | 1/1.9 | 7/63.6 |  |
|  | **Goemai, n=4** | 1/1.9 | - | 3/75 |
|  | **Other*, n=7** | 2/3.8 | 4/36.4 | 1/25 |

*Other - Mwaghavul (3), Tarok (1), Tiv (1), Yoruba (2). Here and other tables, % - of all in this column.

**S2 Table. Patient place of living in different ethnic groups.**

| **Place of living** | **Ethnicity (n/%)** | | | | |
| --- | --- | --- | --- | --- | --- |
|  | **Hausa, n=26** | **Berom, n=22** | **Ngas, n=8** | **Goemai, n=4** | **Other, n=7** |
| **Urban** | 23/88.5 | 22/100 | 2/25 | 3/75 | 2/28.6 |
| **Rural** | 3/11.5 | 0/0 | 6/75 | 1/25 | 5/71.4 |

*Other - Mwaghavul Tarok Tiv Yoruba.

**S3 Table. *M. tuberculosis* drug susceptibility profiles in main genotypes identified in the study.**

| **DST profile** | **Main lineages, (n/%)** | | | | | **Main SITs, (n/%)** | | |
| --- | --- | --- | --- | --- | --- | --- | --- | --- |
|  | **Cameroon,**  **n=42** | **T, n=19** | **LAM, n=2** | **H,**  **n=2** | **L4-unclass.* n=2** | **SIT61, n=33** | **SIT53, n=15** | **SIT17, n=2** |
| **MDR** | 20/47.6 | 11/57.9 | 2/100 | 0/0 | 2/100 | 15/45.5 | 7/46.7 | 2/100 |
| **non-MDR** | 22/52.4 | 8/42.1 | 0/0 | 2/100 | 0/0 | 18/54.5 | 8/53.3 | 0/0 |
| **Monoresistance** | 17/40.5 | 7/36.8 | 0/0 | 2/100 | 0/0 | 12/36.4 | 7/46.7 | 0/0 |

*****L4-unclass. – L4-unclassified; assignment to the Lineage in accordance with an expert assessment.

**S4 Table. Distribution of *M. tuberculosis* main genotypes in the Plateau State.**

| **Genotypes** | | **All, n=67** | **Part of the Plateau State (n/%)** | | |
| --- | --- | --- | --- | --- | --- |
|  |  |  | **North, n=52** | **Center, n=11** | **South, n=4** |
| **Lineages** | **Cameroon** | 42/62.7 | 33/63.5 | 9/81.8 | 0/0 |
|  | **T** | 19/28.3 | 15/28.8 | 1/9.1 | 3/75.0 |
|  | **LAM** | 2/3 | 2/3.9 | 0/0 | 0/0 |
|  | **H** | 2/3 | 1/1.9 | 1/9.1 | 0/0 |
|  | **Other** | 2/3 | 1/1.9 | 0/0 | 1/25 |
| **SITs** | **SIT61** | 33/49.2 | 26/50 | 7/63.6 | 0/0 |
|  | **SIT53** | 15/22.4 | 12/23.1 | 1/9.1 | 2/50 |
|  | **SIT17** | 2/3 | 2/3.9 | 0/0 | 0/0 |
| **Spoligotyping-based HGDI** | | - | 0.704 | 0.618 | NA* |

*NA, non-applicable; too small sample.

**S5 Table. Distribution of *M. tuberculosis* main genotypes in urban *vs*. rural groups.**

| **Genotypes** | | **Place of living (n/%)** | |
| --- | --- | --- | --- |
|  |  | **Urban, n=52** | **Rural, n=15** |
| **Lineages** | **Cameroon** | 33/63.5 | 9/60 |
|  | **T** | 15/28.8 | 4/26.7 |
| **SITs** | **SIT61** | 27/51.9 | 6/40 |
|  | **SIT53** | 12/23.1 | 3/20 |
|  | **SIT17** | 2/3.9 | 0/0 |
| **Spoligotyping-based HGDI** | | 0.684 | 0.828 |

**S6 Table. Distribution of *M. tuberculosis* main genotypes in different ethnic groups.**

| **Genotypes** | | **Ethnicity (n/%)** | | | | | |
| --- | --- | --- | --- | --- | --- | --- | --- |
|  |  | **Hausa, n=26** | **Berom, n=22** | **Ngas,**  **n=8** | **Goemai, n=4** | **Mwaghavul, n=3** | **Other*,**  **n=4** |
| **Lineages** | **Cameroon** | 17/65.4 | 14/63.6 | 5/62.5 | 1/25 | 3/100 | 2/50 |
|  | **T** | 6/23.1 | 8/36.4 | 1/12.5 | 2/50 | 0/0 | 2/50 |
| **SITs** | **SIT61** | 13/50 | 11/50 | 4/50.0 | 1/25 | 3/100 | 1/25 |
|  | **SIT53** | 5/19.2 | 6/27.3 | 1/12.5 | 2/50 | 0/0 | 1/25 |
|  | **SIT17** | 1/3.9 | 0/0 | 1/12.5 | 0/0 | 0/0 | 0/0 |
| **Spoligotyping-based HGDI** | | 0.729 | 0.697 | NA** | NA | NA | NA |

*Other – Tarok, Tiv, Yoruba; **NA, non-applicable; too small sample.

**S7 Table. *M. tuberculosis* drug resistance profiles in main ethnic and religious groups.**

| **DST profile** | **Ethinicity (n/%)** | | | **Place of living (n/%)** | | | **Religious group* (n/%)** | |
| --- | --- | --- | --- | --- | --- | --- | --- | --- |
|  | **Hausa, n=26** | **Berom, n=22** | **Ngas, n=8** | | **Urban, n=52** | **Rural, n=15** | **Moslem,**  **n=26** | **Christian, n=41** |
| **MDR** | 13/50 | 13/59.1 | 4/50 | | 28/53.9 | 7/46.7 | 13/50 | 22/53.7 |
| **Non-MDR** | 13/50 | 9/40.9 | 4/50 | | 24/46.2 | 8/53.3 | 13/50 | 19/46.3 |
| **Monoresistance** | 10/38.5 | 7/31.8 | 4/50 | | 19/36.5 | 7/46.7 | 11/42.3 | 9/22 |

^*^Religious group assigned by ethnicity, i.e. Hausa = Moslem; other = Christian.

**S8 Table. Distribution of main *M. tuberculosis* genotypes in different language families.**

| **Genotypes** | | **Language family (n/%)** | |
| --- | --- | --- | --- |
|  |  | **Afro-Asiatic*, n=41** | **Niger–Congo*, n=26** |
| **Lineages** | **Cameroon** | 26/63.4 | 16/61.5 |
|  | **T** | 9/22.0 | 10/38.5 |
| **SITs** | **SIT61** | 21/51.2 | 12/46.2 |
|  | **SIT53** | 8/19.5 | 7/26.9 |
|  | **SIT17** | 2/4.9 | 0/0 |

*Language family assigned by ethnicity – Hausa, Ngas, Goemai Mwaghavul = Afro-Asiatic; Berom, Yoruba, Tarok, Tiv = Niger–Congo.

**S9 Table. Distribution of main *M. tuberculosis* genotypes in different religious groups.**

| **Genotype** | | **Religious group* (n/%)** | |
| --- | --- | --- | --- |
|  |  | **Moslem, n=26** | **Christian, n=41** |
| **Lineages** | **Cameroon** | 17/65.4 | 25/61.0 |
|  | **T** | 6/23.1 | 13/31.7 |
| **SITs** | **SIT61** | 13/50.0 | 20/48.8 |
|  | **SIT53** | 5/19.2 | 10/24.4 |
|  | **SIT17** | 1/3.9 | 1/2.4 |

^*^Religious group assigned by ethnicity, i.e. Hausa = Moslem; other = Christian.

**S10 Table. Prevalence of the *M. tuberculosis* genotypes in West Africa.**

| **Country** | **No. of isolates; setting; years (Reference)** | **Genotypes and comments** |
| --- | --- | --- |
| Nigeria* | n=81; South (Cross River State); 2008-2009 [1]  n=412; Ibadan Nnewi Abuja; 2009-2010 [2]  n=180; Anambra state; 2009-2011 [3]  n=549; 2013-2014 [4]  n=202; North Central zone of Nigeria (Middle Belt), which includes: Benue, Kogi, Kwara, Nasarawa, Niger, Plateau, and Federal Capital Territory; 2009-2017 (133 in 2017) [5] | SIT61 - 39%; Cameroon - 52%; *M. africanum* -33%; H3 - 5%  SIT61 - 50%; Cameroon - 66%; *M. africanum* 13%;  T2 - 7%; Haarlem 7%; LAM 0.5%; Beijing 0.25%  SIT61 - 51%; Cameroon - 66%; SIT53 - 3%; T - 11%; Afri2 - 11%; H3 - 4.4%  Cameroon - 50% mainly North; SIT61 - 42%;  L5 - 20% (mainly south-east) but absent in 6 states;  L6 - 2.7%; LAM - 1.3%; H3 - 2.4% (incl. SIT49);  T1 - 15% of them SIT53 - 12%; Beijing - 1/549;  L4.6.2/Cameroon more prevalent where *M. africanum* West African 1 (Lineage 5) is absent.  SIT61 - 55%; Cameroon - 63%; Uganda I - 9%; H 5%; L5/WAfr1 3% (further decline of *M. africanum*); LAM - 1.5% |
| Cameroon | n=524; 1997-1998 [6]  n=169; 2009 [7] | *M. africanum* - 6.7%; SIT61 - 32%; Cameroon - 45%; LAM - 2.3%; SIT17 – 2; 0.5% (parental SIT20 - 7); SIT53 - 4%; Haarlem - 15.6%; T - 19%  Cameroon - 34% (all SIT61); Haarlem - 10%; T - 37%; SIT53 -17%; LAM - 1%; *M. africanum* - 1.6% |
| Chad | n=40; 2001-2002 [8]  n=312; 2007-2012 [9] | Cameroon - 33% (13/40); SIT61 - 5/40; SIT53 - 1/40; LAM - 2/40; no *M. africanum;* Lineage 4 - 93%, Lineage 1 - 0.6%, Lineage - 5.7%; Lineage 5 - 0.6%; Haarlem – 5/40 12%; T – 13/40 32%  Cameroon - 41.8%; H1 - 19.2%; T1 - 7.7%; T2 - 6.7%; H3 - 5.4%; T1-RUS2 - 2.8%; X2 - 2.2%; T5-RUS1 - 1.6%.  73 resistant isolates: Cameroon – 35; SIT53 – 6; SIT61 - 20 |
| Benin | n=194; Cotonou; New cases; 2005-2006 [10]  n=100; Retreatment cases; 2014 [11] | SIT61 - 21%; SIT1 – 9%  Cameroon – 46%; ST61 - 33%; T1 – 17%; ST53 - 13%; ST1 - 8%; *M. africanum* – 13%; Haarlem – 6%; LAM – 2% |
| Ghana | n=162; 2007-2009 [12] | *M. tuberculosis* sensu stricto 80%; 20% *M. africanum*; Cameroon - 34%; Haarlem - 12%; Ghana - 17%; Beijing - 3%; EAI - 3%; Uganda I - 3%; LAM - 1.5%; SIT 61 - 25% |
| Ivory Coast | n=194; Retreatment cases [13] | Cameroon – 7.7%; SIT61- 4.1%; T - 75%; SIT53 - 69%; LAM – 5.7%; H - 4.6% (SIT49-2); Beijing - 1.5%; *M. africanum* - 2%. MIRU-VNTR revealed a high level of clustering. |
| Niger | n=222; 2008-2016 [14] | L4 - 92%; Ghana - 47%; Cameroon - 28%; H - 4%; L5 – 2.3%; L6 - 3.6%;  SIT61 – 27%; SIT53 – 46.4% |
| Burkina Faso | n=72; 2010 [15] | Cameroon - 25%; SIT61 - 19%; T - 22%; H - 14%; H3 - 4%; *M. africanum* - 22%; LAM - 1.4%;  SIT53 - 8/72 - 11%; SIT49 - 1/72 |
| Mali | n=492; 2006-2016 [16] | T1 - 32%; Cameroon - 15%; *M. africanum* 2 - 17%; H - 6%; LAM - 6.5%; Beijing -1.8%  SIT53-14%; SIT61-9% |
| Gambia | n=359; 2005-2006 [17] | Mafri – 38%; SIT53 - 8%; Beijing - 2.8%; Haarlem - 14.8%; LAM - 14%; T – 15%; Cameroon – 0.3%; SIT61 – 0.3% |
| Guinea | n=184; 2005-2010 [18] | LAM - 13%; Cameroon - 7%; SIT53 - 11%; SIT61 - 6.5%; *M. africanum* – 5%; Beijing - 4%; Haarlem - 14%; T – 33% |

*only studies published 10 or less years ago were considered.

**REFERENCES**

1. Thumamo BP, Asuquo AE, Abia-Bassey LN, Lawson L, Hill V, Zozio T, et al. Molecular epidemiology and genetic diversity of *Mycobacterium tuberculosis* complex in the Cross River State, Nigeria. Infect Genet Evol. 2012;12: 671-677.
2. Lawson L, Zhang J, Gomgnimbou MK, Abdurrahman ST, Le Moullec S, Mohamed F, et al. A molecular epidemiological and genetic diversity study of tuberculosis in Ibadan, Nnewi and Abuja, Nigeria. PLoS One. 2012;7: e38409.
3. Uzoewulu GN, Lawson L, Nnanna IS, Rastogi N, Goyal M. Genetic diversity of *Mycobacterium tuberculosis* complex strains isolated from patients with pulmonary tuberculosis in Anambra State, Nigeria. Int J Mycobacteriol. 2016;5: 74-79.
4. Molina-Moya B, Gomgnimbou MK, Spinasse L, Obasanya J, Oladimeji O, Dacombe R, et al. *Mycobacterium tuberculosis* complex genotypes circulating in Nigeria based on spoligotyping obtained from Ziehl-Neelsen stained slides extracted DNA. PLoS Negl Trop Dis. 2018;12: e0006242.
5. Pokam BDT, Yeboah-Manu D, Lawson L, Guemdjom PW, Okonu R, Madukaji L, et al. Molecular analysis of *Mycobacterium tuberculosis* isolated in the North Central Zone of Nigeria. J Epidemiol Glob Health. 2019;9: 259-265.
6. Niobe-Eyangoh SN, Kuaban C, Sorlin P, Cunin P, Thonnon J. Genetic biodiversity of *Mycobacterium tuberculosis* complex strains from patients with pulmonary tuberculosis in Cameroon. J Clin Microbiol. 2003;41: 2547–2553.
7. Assam Assam JP, Beng VP, Cho-Ngwa F, Toukam M, Ngoh AAI, Kitavi M, et al. *Mycobacterium tuberculosis* is the causative agent of tuberculosis in the southern ecological zones of Cameroon, as shown by genetic analysis*.* BMC Infect Dis. 2017;13: 431.
8. Diguimbaye C, Hilty M, Ngandolo R, Mahamat HH, Pfyffer GE. Molecular characterization and drug resistance testing of *Mycobacterium tuberculosis* isolates from Chad. J Clin Microbiol. 2006;44: 1575–1577.
9. Ba Diallo A, Ossoga GW, Daneau G, Lo S, Ngandolo R, Djaibé CD, et al*.* Emergence and clonal transmission of multi-drug-resistant tubercul sis among patients in Chad. BMC Infect Dis. 2017;17: 579.
10. Affolabi D, Sanoussi N, Codo S, Sogbo F, Wachinou P, Massou F, et al. First insight into a nationwide genotypic diversity of *Mycobacterium tuberculosis* among previously treated pulmonary tuberculosis cases in Benin, West Africa. Can J Infect Dis Med Microbiol. 2017;2017: 3276240.
11. Affolabi D, Sanoussi N, Codo S, Sogbo F, Wachinou P, Massou F, et al. First insight into a nationwide genotypic diversity of *Mycobacterium tuberculosis* among previously treated pulmonary tuberculosis cases in Benin, West Africa. Can J Infect Dis Med Microbiol. 2017;2017: 3276240.
12. Yeboah-Manu D, Asante-Poku A, Bodmer T, Stucki D, Koram K. Genotypic diversity and drug susceptibility patterns among *M. tuberculosis* complex isolates from South-Western Ghana. PLoS One 6, 2011;2011: e21906.
13. Ouassa T, Borroni E, Loukou GY, Faye-Kette H, Kouakou J. High prevalence of shared international type 53 among *Mycobacterium tuberculosis* complex strains in retreated patients from Cote d’Ivoire. PLoS ONE. 2012;7: e45363.
14. Ejo M, Hassane-Harouna S, Souleymane MB, Lempens P, Dockx J, Uwizeye C, De Rijk P, et al. Multidrug-resistant patients receiving treatment in Niger who are infected with *M. tuberculosis* Cameroon family convert faster in smear and culture than those with *M. tuberculosis*Ghana family. Tuberculosis, 2020;122: 101922.
15. Gomgnimbou MK, Refregier G, Diagbouga SP, Adama S, Kabore A. Spoligotyping of *Mycobacterium africanum*, Burkina Faso. Emerg Infect Dis. 2012;18: 117–119.

in *Mycobacterium tuberculosis* epidemiology. EBioMedicine, 37, 410-416 (2018)

1. Togo ACG, Kodio O, Diarra B, Sanogo M, Coulibaly G, Bane S, et al. The most frequent *Mycobacterium tuberculosis* complex families in Mali (2006-2016) based on spoligotyping. Int J Mycobacteriol. 2017;6: 379-386.
2. de Jong BC, Antonio M, Awine T, Ogungbemi K, de Jong YP, Gagneux S, DeRiemer K, et al. Use of spoligotyping and large sequence polymorphisms to study the population structure of the *Mycobacterium tuberculosis* complex in a cohort study of consecutive smear-positive tuberculosis cases in The Gambia. J Clin Microbiol, 2009;47: 994-1001.
3. Ejo M, Gehre F, Barry MD, Sow O, Bah NM, Camara M, et al. First insights into circulating *Mycobacterium tuberculosis* complex lineages and drug resistance in Guinea*.* Infect Genet Evol. 2015;33: 314-319.
